# Auditory Feedback Assists *Post hoc* Error Correction of Temporal Reproduction, and Perception of Self-Produced Time Intervals in Subsecond Range

**DOI:** 10.3389/fpsyg.2017.02325

**Published:** 2018-01-19

**Authors:** Keita Mitani, Makio Kashino

**Affiliations:** ^1^Department of Information Processing, Interdisciplinary Graduate School of Science and Engineering, Tokyo Institute of Technology, Yokohama, Japan; ^2^Japan Society for the Promotion of Science, Tokyo, Japan; ^3^NTT Communication Science Laboratories, Nippon Telegraph and Telephone Corporation, Atsugi, Japan

**Keywords:** time reproduction, time perception, self-produced timing, auditory feedback, subsecond range, suprasecond range

## Abstract

We examined whether auditory feedback assists the *post hoc* error correction of temporal reproduction, and the perception of self-produced time intervals in the subsecond and suprasecond ranges. Here, we employed a temporal reproduction task with a single motor response at a point in time with and without auditory feedback. This task limits participants to reducing errors by employing auditory feedback in a *post hoc* manner. Additionally, the participants were asked to judge the self-produced timing in this task. The results showed that, in the presence of auditory feedback, the participants exhibited smaller variability and bias in terms of temporal reproduction and the perception of self-produced time intervals in the subsecond range but not in the suprasecond range. Furthermore, in the presence of auditory feedback, the positive serial dependency of temporal reproduction, which is the tendency of reproduced intervals to be similar to those in adjacent trials, was reduced in the subsecond range but not in the suprasecond range. These results suggest that auditory feedback assists the *post hoc* error correction of temporal reproduction, and the perception of self-produced time intervals in the subsecond range.

## Introduction

Timing is essential during various activities, such as performing music and playing sports. In the timing literature, bias and variance in timing tasks have been utilized to construct models of temporal behavior (for review see Repp, 2005; Grondin, 2010; Repp and Su, 2013; Shi et al., 2013a). However, bias and variance can be affected by feedback processing using self-produced timing information. For example, when we play a musical instrument, we perceive the self-produced timing of its sound, and adjust our subsequent motor timing based on sensory information. The quality of the sensory information as regards self-produced timing (i.e., sensory feedback) is sometimes degraded, depending on the external environment. For instance, if we are in a loud environment, information on self-produced timing of auditory inputs are typically degraded. Naturally, we expect such auditory feedback degradation to cause the uncertain perception of self-produced timing and imprecise motor timing. In other words, this implies that auditory feedback assists precise motor timing and the perception of self-produced timing. Nevertheless, the role of such auditory feedback has not been fully understood. An investigation of whether auditory feedback reduces bias and variance in timing tasks provides clues enabling us to precisely model temporal behavior. This study addresses whether or not auditory feedback affects the performance in temporal tasks.

In studies of repetitive tapping, it has been reported that auditory feedback from each tap improves production performance. However, the reported improvement is often small. For example, one study reported that, in a synchronization tapping task with isochronous auditory pacing signals, auditory feedback of the taps reduces the asynchronies between pacing signals and taps but not their variability (Aschersleben and Prinz, 1995). In this study, the reduction in asynchrony by auditory feedback for finger tapping was only 6 ms on average across the participants. In a continuation tapping task without pacing signals, it was reported that auditory feedback reduces the variability of the produced intervals in the subsecond range (Kolers and Brewster, 1985; Chen et al., 2002; Madison and Delignières, 2009), although Chen et al. (2002) reported that the effect of auditory feedback was only 2–3 ms on average across participants in terms of the standard deviation of the produced intervals. In addition to these tapping studies, it was reported that auditory feedback had little or no effect on a well-prepared musical solo performance (Gates and Bradshaw, 1974; Finney, 1997; Repp, 1999; Takahashi and Tsuzaki, 2008). In summary, these studies indicate that immediate auditory feedback plays a small role in precise timing at least in repetitive movement tasks.

Unfortunately, the effects of auditory feedback on temporal reproduction tasks have been poorly investigated. Only one recent study has investigated whether the presence of auditory feedback affects temporal reproduction (Shi et al., 2013b). In the temporal reproduction task employed in this study, the participants attempted to keep a button pressed with or without auditory feedback for the same duration as a previously presented auditory stimulus. They observed a substantial improvement by auditory feedback in terms of bias and variability. They interpreted the result with a reliability based integration model of auditory and motor information to minimize the variance of the produced intervals (see also, Shi et al., 2013a; Shi and Burr, 2016). However, this result could also be involved in multiple processes of error minimization based on auditory feedback. In fact, an observation by Ganzenmüller et al. (2012) suggests two types of error minimization. They manipulated the delay of sensory feedback in a similar task to Shi et al. (2013b). They reported that the delayed onset of auditory feedback immediately lengthens the produced intervals, whereas the delayed offset of auditory feedback gradually shortens them, even though the participants were asked to ignore auditory feedback. Notably, the latter result suggests that automatic *post hoc* error correction is performed by auditory feedback, because the subjective error caused by the delayed offset of auditory feedback cannot be corrected within a trial. However, it remains unclear whether or not immediate auditory feedback assists *post hoc* correction, and whether or not the *post hoc* correction reduces variability of temporal reproduction, because Ganzenmüller et al. (2012) manipulated only the onset and offset delays and did not focus on variability. In another research study, it was found that knowledge of the results (i.e., performance feedback) reduces the variability and/or bias of temporal reproduction (e.g., Aiken, 1965; Montare, 1988; Ryan and Robey, 2002; Ryan, 2016). This result also suggests a *post hoc* error correction mechanism, because the feedback was presented at the end of a trial. Nevertheless, some studies have failed to detect any significant improvement in terms of variability within a participant (e.g., Ryan and Robey, 2002). This implies that error correction does not always lead to a reduction in variability.

**FIGURE 1:**
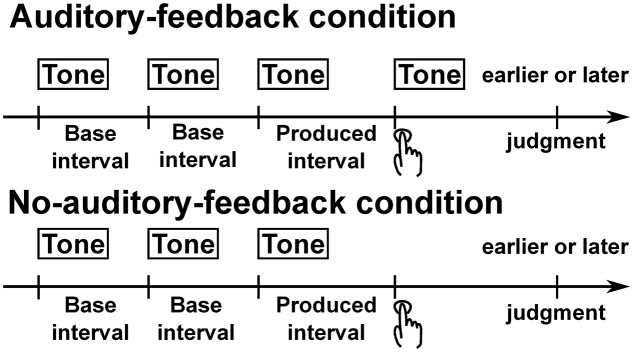
Schematic illustration of trial structure. In the auditory-feedback condition, after participants had listened to three successive tones with two base intervals, they pressed a button to make the interval between the button-press and the last of the three tones subjectively equal to the base interval, and judged whether their button-press was earlier or later than the isochronous timing. In this condition, a feedback tone was immediately presented with the participants’ button-press. In the no-auditory-feedback condition, the task is identical to the auditory-feedback condition. The difference from the auditory-feedback condition is that the feedback tone was not presented.

The remaining questions as regards the role of auditory feedback in temporal reproduction are whether immediate auditory feedback assists *post hoc* error correction, and whether this also reduces the absolute error and variability of the produced intervals. Here, we employed a temporal reproduction task with a single motor response at a point in time (see **Figure 1**). Therefore, unlike the temporal reproduction task used by Shi et al. (2013b), this task does not allow participants to reduce error with auditory feedback within a trial. Thus, this paradigm is suitable for examining the role of auditory feedback in *post hoc* error correction. If auditory feedback has a role in *post hoc* correction, the bias and/or variability in our temporal reproduction task would be reduced by the presence of auditory feedback.

We also analyzed the lag-1 autocorrelation in the temporal reproduction task. This autocorrelation is the correlation between the produced intervals of trials and those of the next trials. A negative value means that the next produced interval of a larger (smaller) produced interval tends to be smaller (larger), suggesting overcorrection of error. Conversely, a positive value means that produced intervals tend to be similar in value to those in adjacent trials, suggesting memorized error and/or small correction. Therefore, this measure provides evidence as to whether or not auditory feedback affects the relationship between adjacent trials. In studies of repetitive tapping, it has been stated that auditory feedback negatively affects the lag-1 autocorrelation of produced intervals (Kolers and Brewster, 1985; Chen et al., 2002). Unlike with repetitive tapping, adjacent produced intervals in temporal reproduction will be less tightly linked to each other. However, we expected auditory feedback to reduce the autocorrelation of produced intervals, when auditory feedback assists *post hoc* error correction.

Moreover, we examined whether the effect of auditory feedback is dependent on target duration in the subsecond and suprasecond ranges. Based on psychophysical, pharmacological, neuroimaging investigations it has been proposed that there are distinct timing mechanisms between these ranges (e.g., Gibbon et al., 1997; Rammsayer, 1999; Lewis and Miall, 2003; Wiener et al., 2010; Grondin, 2012; Hayashi et al., 2014). One class of the hypothesis states that subsecond timing is sensory-automatic whereas suprasecond timing is amodal-cognitive (cf. Rammsayer et al., 2015). This notion is supported by the observation that the difference in temporal sensitivity between auditory and visual modality is more prominent in the subsecond range than in the suprasecond range (Rammsayer et al., 2015). Based on this hypothesis, we expected the effect of auditory feedback to be less in the suprasecond range than in the subsecond range, because a modality-specific mechanism is more involved in the subsecond range (Butler et al., 2011; Kaya et al., 2017). To confirm this, we selected target durations from the subsecond and suprasecond ranges.

Additionally, we measured the variability and bias of the perception of self-produced time intervals in a temporal reproduction task. Although the perception of self-produced time intervals will always accompany temporal reproduction, its properties have only been recently and as yet sparsely explored. Gorea et al. (2010) reported that the perception of self-produced time intervals was worse than that of passively presented time intervals in visual modality and the subsecond range. Mitani and Kashino (2016) reported that the perception of self-produced time intervals was worse than that of passively presented time intervals in the suprasecond range and auditory modality. In contrast to Gorea et al. (2010), Mitani and Kashino (2016) were unable to detect any deterioration, when the target intervals were presented only in the subsecond range. The deterioration in the perception of self-produced timing could be caused by sensory attenuation for self-produced stimuli (for review see Hughes et al., 2013). If this is true, the absence of auditory feedback should cause a substantial deterioration in the perception of self-produced timing in the suprasecond range. However, these previous studies did not focus on the effect of auditory feedback. To clarify this point, in the current study, we examined whether or not auditory feedback improves the perception of self-produced time intervals.

## Materials and Methods

### Participants

Sixteen individuals (5 male, 11 female, age-range 22–47 years) participated in the experiment. All participants had normal hearing. They gave written informed consent and were paid for their participation. This study was conducted in accordance with the Declaration of Helsinki and was approved by the Ethics and Safety Committees of NTT Communication Science Laboratories (Atsugi, Japan). The data for 2 of the 16 participants were excluded because of their reversed psychometric function (see “Analysis” Section). The data obtained from the remaining 14 participants (4 male, 10 female, age range 22–47 years) were analyzed in terms of perception performance.

### Apparatus

The experiment was conducted in a sound-insulated booth. Stimulus presentation and data acquisition were performed by a computer [Apple; Mac Book Air (11 inch, Mid 2013)] using MATLAB 8.1 (The MathWorks) and Psychophysics Toolbox Version 3 (Kleiner et al., 2007). The stimuli were presented through a digital audio interface (Roland; UA-25EX) and headphones (Sennheiser; HDA200). The sampling frequency was 44.1 kHz. The delay between a button press and the presentation of a feedback tone was 23 ± 2 ms [mean ± standard deviation (SD)], as measured by a microphone. In the experiment, this delay was introduced between a button press and auditory feedback. These settings were identical to those of our previous study (Mitani and Kashino, 2016).

### Stimuli and Task

The experiment was conducted under four conditions: 2 (feedback: auditory feedback and no auditory feedback; see **Figure 1**) × 2 (base intervals: 0.5 and 3.2 s). Participants were asked to listen to three successive tones (duration: 50 ms, rise/fall: 10 ms, frequency: 1 kHz, sound pressure level: about 80 dB) with two base intervals, and then immediately press a button (shift key) to create a subjectively isochronous interval between the button-press and the last tone of the three previously presented tones. In the auditory-feedback condition, a feedback tone was presented immediately when the participant pressed the button, whereas in the no-auditory-feedback condition, the feedback tone was not presented. The property of feedback tones was identical with the three successive tones. The participants were also asked to judge whether the produced timing was earlier or later than the subjectively isochronous timing. The judgment was indicated by pressing one of the two buttons (the ← or → key for a judgment of earlier or later, respectively). Except for the first trial of each session, each trial started 1 s after the judgment. The first trial of each session was initiated by the experimenter. The beginning of a trial was indicated by a tone (duration: 50 ms, rise/fall: 10 ms, frequency: 2 kHz, sound pressure level: about 80 dB). After a 6-s silent period from the beginning of a trial, the three successive tones that indicate the base interval were presented.

The participants were asked to use their right index fingers for the temporal reproduction. They were also asked to keep their fingers touching the button, to close their eyes, and not to move their bodies from the beginning of the trials to the reproduction of a base interval. Furthermore, it is known that the mental subdivision of target intervals improves perceptual and motor timing in the suprasecond range, and this would make the difference in the temporal performance of the subsecond and suprasecond ranges unclear (Grondin et al., 2004). Thus, the participants were also asked not to subdivide intervals.

The participants took part in four sessions for each condition. The condition was fixed for each session. The first session for each condition consisted of 24 trials. The data from the first sessions were not analyzed as they were considered to constitute a practice session. The other sessions consisted of 48 trials for each condition. Thus, 144 trials (3 sessions × 48 trials) per condition were analyzed. All the sessions for a given condition were completed, and then the feedback condition was alternated. The base interval condition was alternated after the completion of all the sessions for the two feedback conditions of a base interval condition. The order of the feedback and base interval conditions were counterbalanced across the participants.

### Analysis

First, we excluded the outliers of the produced intervals for each condition and participant. We defined the outliers as below two inter-quartile ranges (IQRs) from the first quartile or above two IQRs from the third quartile.

To evaluate the reproduction performance, we calculated the SD, mean, and lag-1 autocorrelation of the produced intervals for each condition and participant. The autocorrelation was computed in each session, and then averaged across all sessions.

To evaluate perception performance, we estimated the probability function of ‘later’ responses to a produced interval (i.e., psychometric function) in each condition and participant. To estimate the psychometric function, we used a logistic regression undertaken with the maximal likelihood method for the data of pair of judgment and produced interval. The produced interval at a 50% judgment rate was defined as a point of subjective equality (PSE), which indicates the criterion of judgments. Half of the difference between the produced intervals at judgment rates of 25 and 75% was defined as a just noticeable difference (JND), which indicates the variability of judgments. Two participants were excluded from the analysis because their psychometric functions were reversed in at least one condition.

## Results

### Reproduction Performance

**FIGURE 2:**
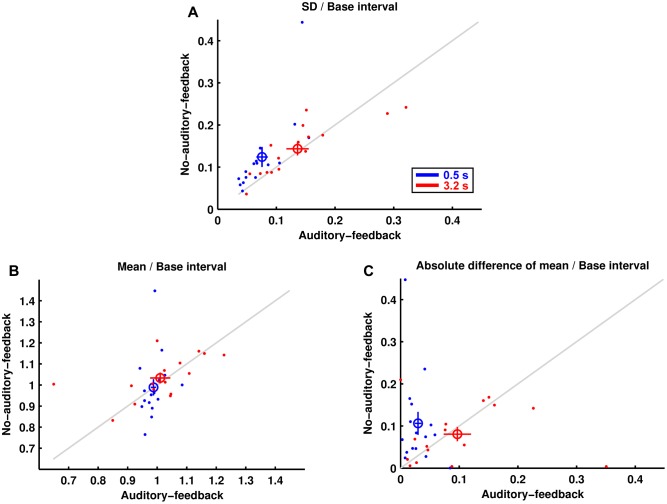
Reproduction performance. **(A)** The SDs of produced intervals divided by their base intervals are represented for each participant by the dots. The colored circles and lines represent the mean across participants and the standard error of the mean, respectively. **(B)** The mean produced intervals divided by their base intervals. **(C)** The absolute differences divided by their base intervals between the mean produced intervals and their base intervals.

**Figure 2A** shows the standard deviations of produced intervals divided by their base interval. The results imply that motor variability for a 0.5-s base interval was less variable in the auditory-feedback condition than in the no-auditory-feedback condition (0.076 ± 0.010, 0.124 ± 0.024, mean ± standard error of mean across participants, in the auditory-feedback and no-auditory-feedback conditions, respectively), whereas that for the 3.2-s base interval was comparable in the feedback conditions (0.136 ± 0.019, 0.143 ± 0.016). A two-way (feedback × base interval) repeated-measures ANOVA indicated a marginally significant main effect of the base interval [*F*(1,15) = 4.53, *p* = 0.050, $\eta_{p}^{2}$ = 0.231], and a significant main effect of feedback [*F*(1,15) = 5.80, *p* = 0.029, $\eta_{p}^{2}$ = 0.279]. More importantly, there was a significant interaction between these effects [*F*(1,15) = 5.74, *p* = 0.030, $\eta_{p}^{2}$ = 0.277]. A *post hoc* analysis of the simple effects suggests that auditory feedback reduced the motor variability in the 0.5-s base interval condition [*F*(1,15) = 7.59, *p* = 0.015, $\eta_{p}^{2}$ = 0.336]. Furthermore, the motor variability of all the participants for the 0.5-s base interval was smaller in the auditory-feedback condition than in the no-auditory-feedback condition. In addition, the motor variability in the auditory-feedback condition was smaller for the 0.5-s base interval than for the 3.2-s base interval [*F*(1,15) = 10.61, *p* = 0.005, $\eta_{p}^{2}$ = 0.414]. The advantage of subsecond timing in an auditory modality that we observed is consistent with previous studies (e.g., Grondin, 2012; Mitani and Kashino, 2016). However, this advantage was not detected in the no-auditory-feedback condition [*F*(1,15) = 0.74, *p* = 0.403, $\eta_{p}^{2}$ = 0.047].

**Figure 2B** shows the mean produced interval divided by its base interval for each condition. The results imply that the mean produced intervals in the auditory-feedback condition and the no-auditory-feedback condition were comparable for a 0.5-s base interval (0.987 ± 0.009, 0.989 ± 0.039) and for a 3.2-s base interval (1.010 ± 0.034, 1.033 ± 0.025). The ANOVA indicated no significant effect [feedback: *F*(1,15) = 0.39, *p* = 0.540, $\eta_{p}^{2}$ = 0.026, base interval: *F*(1,15) = 1.37, *p* = 0.260, $\eta_{p}^{2}$ = 0.084, interaction: *F*(1,15) = 0.16, *p* = 0.695, $\eta_{p}^{2}$ = 0.011].

**Figure 2C** shows the absolute difference divided by its base interval between the mean produced interval and its base interval. A smaller value of this measure means a smaller motor bias. The results imply that the motor bias for the 0.5-s base interval was smaller in the auditory-feedback condition than in the no-auditory-feedback condition (0.030 ± 0.006, 0.106 ± 0.027), whereas that for the 3.2-s base interval was comparable in the feedback conditions (0.097 ± 0.023, 0.081 ± 0.017). The ANOVA indicated a significant interaction [*F*(1,15) = 4.78, *p* = 0.045, $\eta_{p}^{2}$ = 0.241]. The main effects of feedback [*F*(1,15) = 2.53, *p* = 0.132, $\eta_{p}^{2}$ = 0.145] and base interval [*F*(1,15) = 1.06, *p* = 0.320, $\eta_{p}^{2}$ = 0.066] were not significant. The results of a *post hoc* analysis indicated that auditory feedback reduced the motor bias in the 0.5-s base interval condition [*F*(1,15) = 6.80, *p* = 0.020, $\eta_{p}^{2}$ = 0.312], and the motor bias in the auditory-feedback condition was smaller for the 0.5-s base interval than for the 3.2-s base interval [*F*(1,15) = 7.68, *p* = 0.014, $\eta_{p}^{2}$ = 0.339]. Similar to the variability of temporal reproduction, the advantage of subsecond timing was not detected in the no-auditory-feedback condition [*F*(1,15) = 0.58, *p* = 0.458, $\eta_{p}^{2}$ = 0.037].

### Perception Performance

**FIGURE 3:**
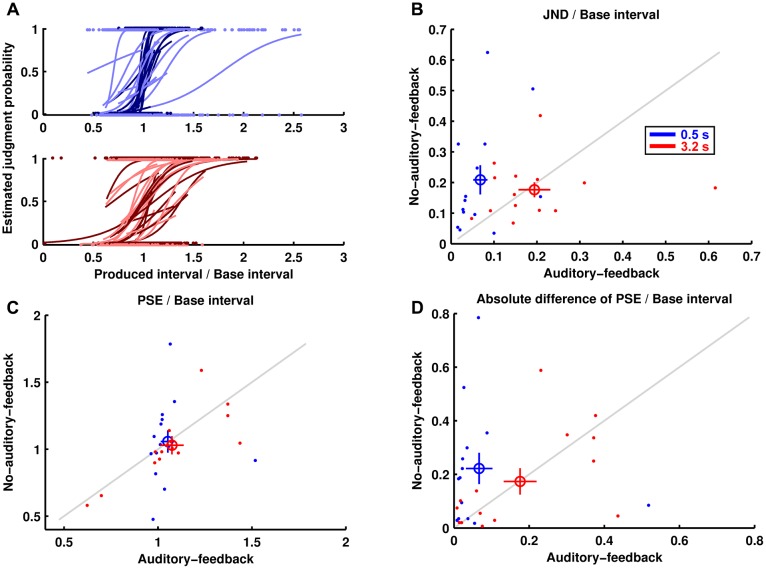
Perception performance. **(A)** The estimated psychometric functions and the data for pairs consisting of produced interval and judgment of the 14 participants are displayed. The darker and lighter colors indicate auditory feedback and no auditory feedback conditions, respectively. **(B)** The JNDs divided by their base intervals are displayed in the same manner as the reproduction performance. **(C)** The PSEs divided by their base intervals. **(D)** The absolute differences divided by their base intervals between the PSEs and their base intervals.

**Figure 3A** shows the estimated psychometric functions of the 14 participants. In general terms, the perceptual performance results were similar to the motor performance results. **Figure 3B** shows the JNDs divided by their base interval (i.e., perceptual variability). The results imply that the perceptual variability for the 0.5-s base interval was lower in the auditory-feedback condition than in the no-auditory-feedback condition (0.068 ± 0.017, 0.209 ± 0.048), whereas that for the 3.2-s base interval was comparable in the feedback conditions (0.194 ± 0.037, 0.176 ± 0.024). The ANOVA indicated a marginally significant main effect of feedback [*F*(1,13) = 3.67, *p* = 0.078, $\eta_{p}^{2}$ = 0.220] and a significant interaction [*F*(1,13) = 6.81, *p* = 0.022, $\eta_{p}^{2}$ = 0.344]. The main effect of the base interval was not significant [*F*(1,13) = 1.63, *p* = 0.224, $\eta_{p}^{2}$ = 0.112]. A *post hoc* analysis of the simple effects suggests that auditory feedback reduced the perceptual variability in the 0.5-s base interval condition [*F*(1,13) = 10.12, *p* = 0.007, $\eta_{p}^{2}$ = 0.438]. In addition, the perceptual variability in the auditory-feedback condition for the 0.5-s base interval was smaller than for the 3.2 s base interval [*F*(1,13) = 9.79, *p* = 0.008, $\eta_{p}^{2}$ = 0.430]. As with the reproduction performance, the advantage of subsecond timing was not detected in the no-auditory-feedback condition [*F*(1,13) = 0.32, *p* = 0.581, $\eta_{p}^{2}$ = 0.024].

**Figure 3C** shows the PSEs divided by their base interval. The results imply that PSE in the auditory-feedback condition was comparable to that in the no-auditory-feedback condition for the 0.5-s base interval (1.052 ± 0.037, 1.057 ± 0.083) and for the 3.2-s base interval (1.075 ± 0.062, 1.029 ± 0.068). The ANOVA indicated no significant effects [feedback: *F*(1,13) = 0.156, *p* = 0.700, $\eta_{p}^{2}$ = 0.012, base interval: *F*(1,13) = 0.002, *p* = 0.963, $\eta_{p}^{2}$ = 0.000, interaction: *F*(1,13) = 0.276, *p* = 0.608, $\eta_{p}^{2}$ = 0.021].

**Figure 3D** shows the absolute difference divided by its base interval between the PSEs and their base interval (i.e., perceptual bias). The results imply that the perceptual bias for the 0.5-s base interval was less in the auditory-feedback condition than in the no-auditory-feedback condition (0.067 ± 0.035, 0.222 ± 0.058), whereas that for the 3.2-s base interval was comparable in the feedback conditions (0.176 ± 0.044, 0.174 ± 0.049). However, the ANOVA indicated only a marginally significant main effect of feedback [*F*(1,13) = 3.70, *p* = 0.077, $\eta_{p}^{2}$ = 0.222] and the interaction [*F*(1,13) = 3.33, *p* = 0.091, $\eta_{p}^{2}$ = 0.204]. The main effect of the base interval was not significant [*F*(1,13) = 0.30, *p* = 0.595, $\eta_{p}^{2}$ = 0.022].

### Autocorrelation

**FIGURE 4:**
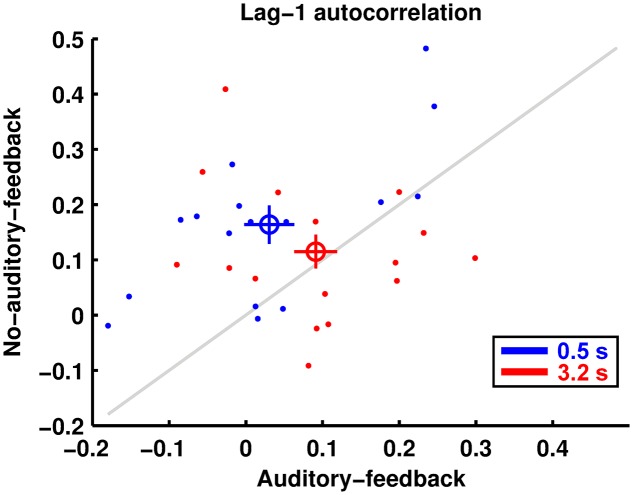
The lag-1 autocorrelations of produced intervals are displayed in the same manner as the reproduction and perception performances.

**Figure 4** shows the lag-1 autocorrelation of the produced intervals. The results imply that the lag-1 autocorrelation for the 0.5-s base interval was lower in the auditory-feedback condition than in the no-auditory-feedback condition (0.030 ± 0.033, 0.164 ± 0.035), whereas that for the 3.2-s base interval was comparable in the two conditions (0.091 ± 0.028, 0.115 ± 0.031). The ANOVA indicated a significant main effect of feedback [*F*(1,15) = 7.86, *p* = 0.013, $\eta_{p}^{2}$ = 0.344] and a significant interaction [*F*(1,15) = 4.74, *p* = 0.046, $\eta_{p}^{2}$ = 0.240]. The main effect of the base interval was not significant [*F*(1,15) = 0.03, *p* = 0.858, $\eta_{p}^{2}$ = 0.002]. A *post hoc* analysis of the simple effects suggests that auditory feedback reduced the lag-1 autocorrelation in the 0.5-s base interval condition [*F*(1,15) = 24.02, *p* = 0.0002, $\eta_{p}^{2}$ = 0.616]. Furthermore, a single sample *t*-test indicates that for all conditions except the auditory-feedback and 0.5-s base interval condition these indexes were significantly positive [in the auditory-feedback condition, 0.5-s: *t*(15) = 0.93, *p* = 1, Cohen’s *d* = 0.233, 3.2-s: *t*(15) = 4.71, *p* = 0.001, Cohen’s *d* = 1.178, in the no-auditory-feedback condition, 0.5-s: *t*(15) = 3.26, *p* = 0.02, Cohen’s *d* = 0.815, 3.2-s: *t*(15) = 3.74, *p* = 0.008, Cohen’s *d* = 0.934, these *p*-values were corrected by the Bonferroni method]. This result implies that temporal reproduction in a trial is performed with a produced interval memorized in the previous trial.

## Discussion

We investigated whether auditory feedback affects the performance of temporal reproduction with a single motor response at a point in time, and the perception of self-produced time intervals in the subsecond and suprasecond ranges. The results indicated that auditory feedback improves both temporal reproduction and the perception of self-produced time intervals in the subsecond range but not in the suprasecond range. The results also indicated that auditory feedback reduces the serial dependency (i.e., lag-1 autocorrelation) of temporal reproduction in the subsecond range but not in the suprasecond range. Furthermore, we found that the serial dependency was significantly positive in all conditions except under the auditory-feedback and 0.5-s base interval conditions. In addition, the substantial deterioration in the perception of self-produced timing caused in absence of auditory feedback was limited to the subsecond range. This result clearly indicates the invalidity of the sensory attenuation explanation for the deteriorated perception of self-produced timing in the suprasecond range.

The reduced variability and bias of temporal reproduction and the perception of self-produced time intervals by auditory feedback are consistent with previous studies indicating that the perception of intermodal intervals is less stable than that of intramodal intervals (for review see Grondin, 2014). In our study, the auditory-feedback condition participants judged the intramodal interval in an auditory modality, while in the no-feedback condition they judged the intermodal interval between an auditory and a somatosensory modality. Our results show that the advantage of an intramodal interval is valid in a condition where the self-produced intervals are intramodal or intermodal in the perceptual and motor timing of the subsecond range.

This improvement caused by auditory feedback in the subsecond range must be involved in the stabilization of the internal representation of self-produced time intervals by auditory feedback. There are several possible reasons for this stabilization. One possibility is the additional noise originating from the translation of a modal representation into an amodal representation to calculate the intermodal interval, when auditory feedback is not provided. This consideration is based on the notion that modality-specific and amodal mechanisms underlie the processing of subsecond timing. Another possibility is multisensory integration, which can be considered another aspect of amodal mechanisms. It has been suggested that human beings combine information from multisensory modalities to reduce the variability of estimates based on the reliabilities of each multisensory modality in time perception (for review see Shi et al., 2013a; Shi and Burr, 2016). In our paradigm, temporal information regarding auditory and somatosensory modalities can be integrated in the auditory-feedback condition, whereas it cannot be integrated in the no-auditory-feedback condition. Moreover, there are other possibilities, including subjectively indefinite temporal correspondence between sensory and motor modalities (e.g., Fujisaki et al., 2004; Vroomen et al., 2004; Stetson et al., 2006). Although further investigations are needed to estimate the precise nature underlying the stabilization of the representation of self-produced time intervals by auditory feedback in the subsecond range, the assumption that a modality-specific mechanism underlies subsecond timing is needed to explain this stabilization.

The comparable variability of temporal reproduction and the perception of self-produced time intervals in the feedback conditions in the suprasecond range does not necessarily mean that auditory feedback has no effect in the suprasecond range. It can be considered that there are duration-dependent and duration-independent noises for motor and perceptual timing (Ivry and Hazeltine, 1995; Merchant et al., 2008). If the effect of auditory feedback is independent of target duration, this must be concealed by duration-dependent noise. In our experiment, the average difference in the SD of the produced intervals between the feedback conditions in the subsecond range was about 4.8% of 0.5 s. Although the difference is critical for subsecond timing, in the suprasecond range it is relatively small compared with the base interval. The reason is that 24 ms (4.8% of 0.5 s) is less than 1% of 3.2 s. If the same difference exists in the suprasecond range as in the subsecond range (i.e., the effect of auditory feedback is duration-independent), we could not detect it. Thus, we cannot conclude that auditory feedback has no effect in the suprasecond range. However, we can conclude that the improvement due to auditory feedback is at least negligible compared with the amount of variability in the suprasecond range based on the current behavioral data. Taken together with the substantial improvement effect of auditory feedback on subsecond timing, this result mainly supports the hypothesis that subsecond timing is sensory-automatic and suprasecond timing is amodal-cognitive.

To reduce the variability and bias of temporal reproduction, the representation of self-produced time intervals must be stabilized and the temporal reproduction must be performed using the stabilized representation. The reduced lag-1 autocorrelation with auditory feedback in the subsecond range supports this idea. When participants know that a produced interval in a trial is overproduced, they will try to make the next produced interval shorter than the previous one. Therefore, error correction must lead to the reduction of the lag-1 autocorrelation of produced intervals.

In addition, the positive lag-1 autocorrelation of produced intervals was found in all conditions except the auditory-feedback and subsecond conditions. This result implies that the self-produced time interval information is used not only when auditory feedback is available but also when it is not available. This idea is plausible because computing for temporal reproduction with the memory of previous intervals will lead to a greater reduction in variability and more computational loss than that without this memory. Consistent with this idea, Wiener et al. (2014) successfully reproduced their participants’ bias and variability of time perception induced by a temporal context with a model using the memory of previous intervals. Nevertheless, the positive lag-1 autocorrelation will be found in a presumed timing system that does not use information about the self-produced time intervals. For example, participants’ internal states, such as arousal, must be similar in adjacent trials, and these similar internal states will lead to similar produced intervals. Therefore, further study will be needed to dissociate these possibilities.

## Conclusion

We examined the role of auditory feedback in temporal reproduction, and in the perception of self-produced intervals. The current study demonstrated that auditory feedback assists *post hoc* error correction in temporal reproduction, and reduces the variability and bias of temporal reproduction and the perception of self-produced time intervals in the subsecond range. Our findings provide an insight how sensory feedback contributes to reducing the errors of motor and perceptual timing. We consider that they will contribute to the precise modeling of temporal processing.

## Author Contributions

KM and MK conceived and designed the study. KM performed testing, data collection and data analysis. KM drafted the paper. MK provided critical revisions. KM and MK approved the final version of the paper for submission.

## Conflict of Interest Statement

MK is an employee of Communication Science Laboratories, which is a basic-science research section of Nippon Telegraph and Telephone Corporation. The other author declares that the research was conducted in the absence of any commercial or financial relationships that could be construed as a potential conflict of interest.
